# *Salmonella enterica subspecies arizonae* infection of adult patients in Southern Taiwan: a case series in a non-endemic area and literature review

**DOI:** 10.1186/s12879-016-2083-0

**Published:** 2016-12-09

**Authors:** Yi-Chien Lee, Miao-Chiu Hung, Sheng-Che Hung, Hung-Ping Wang, Hui-Ling Cho, Mei-Chu Lai, Jann-Tay Wang

**Affiliations:** 1 Department of Internal Medicine, Ditmanson Medical Foundation Chia-Yi Christian Hospital, Chia-Yi, Taiwan; 2 Department of Pediatrics, Taipei Veterans General Hospital, Taipei, Taiwan; 3 Department of Radiology, Taipei Veterans General Hospital, Taipei, Taiwan; 4 School of Medicine, National Yang-Ming University, Taipei, Taiwan; 5 Department of Nursing, Ditmanson Medical Foundation Chia-Yi Christian Hospital, Chia-Yi, Taiwan; 6 Department of Laboratory Medicine, Ditmanson Medical Foundation Chia-Yi Christian Hospital, Chia-Yi, Taiwan; 7 Department of Internal Medicine, National Taiwan University Hospital, No. 7 Chung-Shan South Road, Taipei, 10002 Taiwan

**Keywords:** *Salmonella arizonae*, Peptic ulcer disease, Proton pump inhibitors, Third generation cephalosporins, Ampicillin

## Abstract

**Background:**

The majority of *Salmonella arizonae* human infections have been reported in southwestern United States, where rattlesnake-based products are commonly used to treat illness; however, little is known in non-endemic areas. We reviewed and analyzed the clinical manifestations and treatment outcomes in adult patients with *S. arizonae* infection at our institution.

**Method:**

A retrospective study was conducted at a regional teaching hospital in southern Taiwan from July 2007 to June 2014. All adult patients diagnosed with *S. arizonae* infections and treated for at least three days at Chia-Yi Christian Hospital were included. Patients were followed till discharge.

**Results:**

A total of 18 patients with *S. arizonae* infections (median age: 63.5 years) were enrolled for analysis, of whom two thirds were male. The three leading underlying diseases were diabetes mellitus, peptic ulcer disease and malignancy. Ten patients had bacteraemia and the most common infection focus was the lower respiratory tract. Most of the patients (72.2%) received third-generation cephalosporins as definitive therapy. In contrast, ampicillin-based regimens (accounting for 45.2%) were the major treatment modalities in previous reports. The crude in-hospital mortality was 5.6%, which was much lower than what was previously reported (22.7%).

**Conclusions:**

Though uncommon, there were cases of *S. arizonae* infections in Taiwan. Patients receiving third-generation cephalosporins treatment had better prognosis compared with those treated with ampicillin-based regimen.

## Background

*Salmonellae* are Gram-negative, non-spore-forming, facultatively anaerobic bacilli belonging to the family of *Enterobacteriaceae*, which usually cause food-borne diseases. *Salmonella arizonae*, one of the less common members of *Salmonellae*, has the distinguishing biochemical characteristics of the ability to ferment lactose, utilize malonate, liquefy gelatin, and inhibition by the presence of potassium cyanide [1]. It was first reported in diseased reptiles in 1939 by Caldwell and Ryerson, and was named *Salmonella dar-es-salaam* at that time [2]. It has also been subsequently named *Arizona hinshawii*, *S. arizonae*, *S. cholerasuis* subsp. *arizonae* and finally reclassified as *S. enterica* subsp. *arizonae* in 1983 (*S. arizonae* was used throughout this manuscript) [3].

It was initially considered to be pathogenic only in reptiles, especially in snakes, with as many as 78.8% of them harboring the organism [4]. It was occasionally responsible for severe outbreaks in turkeys and sheep [4]. The first case of human infection by *S. arizonae*, presented with gastroenteritis, was recognized in 1944 [5]. Thereafter, *S. arizonae* was noted to be able to cause a spectrum of human diseases, including gastroenteritis, bacteraemia, vascular infection, bone and joint infection, and central nervous system infection [1, 4, 6–22]. Most of these infections have a good prognosis without any complications. However, severe human infection caused by *S. arizonae* has been documented in children below 7 years of age [23], immunocompromised adults, (e.g., autoimmune diseases under steroid therapy [1, 8, 10, 11, 14, 24, 25], malignancy [7, 13, 14, 21, 26–29], human immunodeficiency virus (HIV) infection [1, 15, 17, 19, 30, 31], organ transplantation [11, 32]), or even in immunocompetent populations [18].

Cold-blooded animals are the usual habitats of *S. arizonae*, especially in reptiles or rattlesnake-based products. Other animals, including poultry, rats, and dogs [6, 9, 17], have also been involved in human infection by *S. arizonae*. According to previously published articles [1, 10, 13, 14, 16, 25–29, 31], the geographic distributions of *S. arizonae* human infections are mainly located in south-western United States, where the use of rattlesnake-associated products to treat a wide variety of illnesses is popular among Mexican-Americans populations. Around 20 pediatric patients below 18 years of age suffering from *S. arizonae* infection have been reported in the literature [9, 17, 21, 23]. Little is known about *S. arizonae* infection of adult patients in Asian countries, including Taiwan. The aim of this study was to analyze all adult patients with *S. arizonae* infection at a regional teaching hospital in southern Taiwan and to perform a literature review on similar patients.

## Methods

From July 2007 to June 2014, all adult patients (≥18 years) diagnosed with *S. arizonae* infection and treated for at least 3 days at Chia-Yi Christian Hospital (CYCH, a regional teaching hospital with a capacity of 1000 beds in southern Taiwan) were retrospectively enrolled. *S. arizonae* infection was defined as positive cultures of any kind of clinical specimens, including blood, pleural effusion, ascites, urine, sputum, stool, pus and bone, for *S. arizonae* plus the presence of signs of systemic or local infection. All types of specimens were collected, transported, and processed according to the suggestion described previously [33], and then inoculated into the corresponding culture media for subsequent incubation [34]. The blood culture system at CYCH was Bactec FX system (Becton, Dickinson and Co. [BD], Franklin Lakes, NJ). All bone tissues were collected by bone biopsy or surgical procedures. Potential pathogens were identified by Vitek version 2.0 (bio Merieux Suisse S.A., Geneva, Switzerland), and *Salmonella* isolates were confirmed by serologic testing (Difco^TM^ Antiserum Solutions). The culture-positive cases were identified by reviewing microbiology records at CYCH.

A standardized case report form was used to collect the demographics, clinical and laboratory data and treatment outcomes. Patients who used H_2_-receptor antagonists or proton pump inhibitors within one month prior to admission were defined as having peptic ulcer disease. Leukocytosis was defined as white blood cell counts exceeding 10 K/μl and thrombocytopenia was defined as platelet count below 150 K/μl. The infection foci of non-bacteraemic patients and patients with secondary bacteraemia were determined if there was a presence of clinical symptoms or signs of infection and isolation of *S. arizonae* from related clinical specimens. Bacteraemia without an obvious infection source or related to intravascular catheter infection or vascular lesions was classified as primary bacteraemia.

The antimicrobial susceptibilities to chloramphenicol, ciprofloxacin, trimethoprim/sulfamethoxazole, ampicillin and ceftriaxone were determined using the disk diffusion method according to the recommendation of the Clinical and Laboratory Standards Institute (CLSI) [35]. The results were also interpreted using the criteria suggested by CLSI [36]. Antimicrobial agents given before the susceptibility results were defined as empirical therapy, whereas definitive therapy was defined as effective antibiotic therapy prescribed according to the results of final blood cultures and susceptibility testing. The study was approved by the ethics review boards of the hospital [Chia-Yi Christian Hospital-Institutional Review Board (CYCH-IRB) No. 104035, 06/30/2015]. The IRB waived informed consent due to the retrospective study design and the research posing no more than minimal risk.

Continuous variables were described as medians with interquartile ranges (IQR) and categorical variables were described as percentage.

## Results

A total of 485 adult patients with *Salmonella* species infection were identified during this seven-year period, and only 4.7% (23/485) of those patients suffered from *S. arizonae* infection. Five among them were excluded because they received treatment for less than 3 days at CYCH and did not follow-up subsequently. For the five eliminated patients, the median age was 65 years (IQR, 60–72 years). Four of them had primary bacteraemia and one had pneumonia. The demographics, clinical features, laboratory data and treatment outcome of the enrolled 18 patients are showed in Table 1. Of these 18 patients, the median age was 63.5 years, ranging from 27 to 81 years, with 12 men and six women. All patients lived in Chiayi City, except one, who lived in Yunlin County. All patients had various underlying diseases, including endocrine diseases in 12 (66.7%; diabetes mellitus in ten, and another diseases in two), peptic ulcer disease in eight (44.4%), malignancy in eight (34.8%; hepatocellular carcinoma in four, lung cancer in three, and colon cancer in one), hypertension in seven (38.9%), liver cirrhosis in six (33.3%), chronic viral hepatitis in six (33.3%; hepatitis B virus in five, and hepatitis C virus in two), chronic kidney disease in three (16.7%), autoimmune diseases in one, and acquired immunodeficiency syndrome in one (5.6%).

**Table 1:** Demographics and clinical data of 18 patients with *S. arizonae* infections in our case series

Age by years (median, range)	63.5 (27–81)
Male to female ratio	12 : 6
Underlying diseases (No., %)	
Diabetes mellitus	10 (55.6)
Hypothyroidism	2 (11.1)
Peptic ulcer diseases	8 (44.4)
Hepatocellular carcinoma	4 (22.2)
Lung cancer	3 (16.7)
Colon cancer	1 (5.6)
Multiple myeloma	1 (5.6)
Hypertension	7 (38.9)
Liver cirrohosis	6 (33.3)
Hepatitis B	5 (27.8)
Hepatitis C	2 (11.1)
Chronic kidney diseases	3 (16.7)
Systemic lupus erythematosus	1 (5.6)
AIDS	1 (5.6)
Symptoms (No., %)	
Fever	9 (50.0)
Abdominal discomfort	7 (38.9)
Dyspnea	7 (38.9)
Laboratory data (No., %)	
WBC > 10000/μl	10 (55.6)
CRP > 10 mg/dL	8/17 (47.1)
ALT > 44 U/L	8/17 (47.1)
Creatinine > 1.3 mg/dl	7/17 (41.2)
Site of bacterial isolation (No., %)	
Blood	10 (55.6)
Bone	2 (11.1)
Abscess	2 (11.1)
Pleural effusion	2 (11.1)
Sputum	1 (5.6)
Stool	1 (5.6)
Ascites	1 (5.6)
Urine	1 (5.6)
Clinical presentations (No., %)	
Lower respiratory tract infection	4 (22.2)
Bone and joint infection	3 (16.7)
Gastrointestinal tract infection	3 (16.7)
Intra-abdominal infection/peritonitis	3 (16.7)
Soft tissue infection	2 (11.1)
Mycotic aneurysm	2 (11.1)
Urinary tract infection	1 (5.6)
Definite antibiotic treatment (No., %)	
3^rd^ generation cephalosporins	13 (72.2)
Fluoroquinolones	3 (16.7)
Piperacillin/tazobactam	1 (5.6)
TMP/SMX	1 (5.6)
Intensive care unit stay (No., %)	9 (50.0)
Length of hospitalization, days (mean ± standard deviation)	19.7 ± 13.7
In-hospital mortality (No., %)	1 (5.6)

*AIDS* acquired immunodeficiency syndrome, *WBC* white blood cell, *CRP* C-reactive protein, *ALT* alanine aminotransferase, *TMP/SM* trimethoprim/sulfamethoxazole

Nine patients (50.0%) had fever as their first presentations. The other initial presentations included abdominal discomfort (7/18, 38.9%), dyspnoea (7/18, 38.9%), cough (3/18, 16.7%), diarrhea (3/18, 16.7%), change in consciousness (2/18, 11.1%), and arthralgia (2/18, 11.1%). The median initial body temperature was 37.2 °C (IQR, 36.1–38.3) and 14 patients (77.8%) had initial heart rates above 90 beats per minute. Hypotension (systolic blood pressure < 90 mmHg) occurred in three patients. Leucocytosis was noted in ten patients (55.6%), and nine (50.0%) patients had thrombocytopenia. Seventeen patients had neutrophilia and eight patients (47.1%, one missing data) had elevated C-reactive protein levels of up to more than 10 mg/dL. For liver function tests, 11 patients (64.7%) had elevated aspartate or alanine transaminase values (AST > 38 U/L or ALT > 44 U/L). Impaired renal function at presentation, defined as serum creatinine > 1.3 mg/dl, was noted in 41.2% (7/17, one missing data) of patients. Initial serum albumin level was available in 13 patients only and 11 of them (84.6%) had hypoalbuminemia (albumin < 3 g/dL). Hyponatremia (sodium < 135 mmol/L) was found in 82.4% of patients (14/17, one missing data), and only four patients (23.5%, one missing data) had hypokalaemia (potassium < 3.5 mmol/L). Serum glucose level at presentation was only available in 13 patients, and five (38.5%) of them had elevated levels of ≥ 200 mg/dL.

Blood cultures were obtained in all 18 patients, and bacteraemia was identified in ten (55.6%), including primary bacteraemia in two, both with mycotic aneurysm and secondary bacteraemia in eight. The most common infection foci of patients with secondary bacteraemia and non-bacteraemic clinical syndromes were lower respiratory tract (4/18, 22.2%), followed by bone and joint (*n* = 3, 16.7%), gastrointestinal tract (*n* = 3, 16.7%), intra-abdominal infection or peritonitis (*n* = 3, 16.7%), soft tissue (*n* = 2, 11.1%), and urinary tract (*n* = 1, 5.6%). In-vitro susceptibility testing revealed that all isolates were susceptible to all of the five tested antimicrobials, i.e. no strains had decreased susceptibility to ciprofloxacin according to the 2013 CLSI guidelines. Seven patients received effective empirical antibiotics. The definite therapy included third-generation cephalosporins in 13 (72.2%) of 18 patients, of which three patients received combined therapy (oral co-trimoxazole in two and ciprofloxacin in one), fluoroquinolone in three (16.7%), piperacillin/tazobactam in one (5.6%), and oral co-trimoxazole in one (5.6%). The median duration of treatment with parenteral antibiotics was 12 days, ranging from 5 to 62 days. Of the 18 patients, the median hospital stay was 14 days, ranging from 6 to 62 days. Nine (50.0%) patients were admitted to the intensive care unit during their hospitalization. 17 patients recovered from this infection successfully after completion of the treatment course, and one patient died. Overall, the crude in-hospital mortality rate was 5.6% (1/18).

## Discussion

Our present study demonstrates that *S. arizonae* infection is uncommon among adult patients with a crude in-hospital mortality rate of 5.6%. To the best of our knowledge, this is the largest case series reporting adult patients infected by *S. arizonae*. Based on a thorough search in PubMed, there were 27 studies reporting and discussing patients with *S. arizonae* infection from 1959 to the writing of the present manuscript. Clinical characteristics of these 44 reported patients, including age, gender, underlying diseases, type of infection, antibiotic therapy, exposure and treatment outcome, are displayed in Table 2.

**Table 2:** Characteristics, clinical diseases and outcome of 44 human infections with *S. arizonae* reported in the literature (1959–2012)

Reference	Age	Gender	Underlying Diseases	Type of Infection	Antibiotic Treatment	Exposure	Outcome
Krag 1959 [20]	63	F	idiopathic thrombocytopenic purpura	knee septic arthritis	achromycin	unknown	death
Guckian 1967 [24]	52	F	SLE, DM	knee septic arthritis/gastroenteritis/UTI	cephalothin/ampicillin/chloramphenicol	unknown	recover
Andrews 1970 [6]	21	M	none	gastroenteritis	ampicillin/chloramphenicol	dog	recover
Smilack 1975 [8]	23	F	SLE	knee/shoulder arthritis/tibial abscess	ampicillin/cephalothin	unknown	recover
Arora 1976 [18]	30	M	none	bacteraemia	chloramphenicol	unknown	recover
Keren 1976 [12]	53	M	alcoholism	osteomyelitis/gastroenteritis	ampicillin/aminoglycoside	unknown	recover
Johnson 1976 [32]	29	M	Hodgkin’s disease	bacteraemia/gastroenteritis/UTI	ampicillin	raw milk	death
Johnson 1976 [32]	54	M	post heart transplant	bacteraemia/gastroenteritis	cephalothin/gentamicin	unknown	death
Johnson 1976 [32]	54	F	mental retardation	gastroenteritis/pneumonia/empyema	ampicillin	unknown	recover
Petru 1981 [22]	53	M	DM/interstitial pneumonitis	bacteraemia/mycotic aneurysm	ampicillin/gentamicin	NA	recover
Lindsay 1981 [40]	69	F	cirrhosis	bacteraemia/peritonitis	cephalosporin/aminoglycoside/chloramphenicol	unknown	death
Fainstein 1982 [13]	66	M	leukaemia	bacteraemia	ticarcillin/aminoglycoside	rattlesnake capsule	death
Mcintyre 1982 [16]	73	M	DM/HTN	septic arthritis/aortic aneurysm/UTI	ampicillin	rattlesnake capsule	recover
CDC 1983 [7]	63	F	gallbaldder adenocarcinoma	bacteraemia	?	Rattlesnake capsule	recover
Quismorio 1983 [11]	31	F	SLE	septic arthritis	ampicillin	unknown	death
Quismorio 1983 [11]	41	M	HBV, post renal transplant	septic arthritis/kidney abscess	ampicillin	unknown	death
Quismorio 1983 [11]	48	F	Waldenstrom macroglobulinemia	septic arthritis/gastroenteritis/UTI	ampicillin	unknown	death
Riley 1988 [1]	19	F	SLE	bacteraemia	ampicillin	rattlesnake capsule	recover
Riley 1988 [1]	25	M	AIDS	adenitis/empyema	TMP/SMX	rattlesnake capsule	recover
Riley 1988 [1]	51	M	HTN/DCM/CHF/atrial fibrillation	pleurisy	nil	rattlesnake capsule	recover
Bhatt 1989 [10]	27	F	SLE	bacteraemia/gastroenteritis/peritonitis/ meningitis	ampicillin	rattlesnake meat	death
Fleischman 1989 [28]	38	M	gastric carcinoma	bacteraemia/empyema	ampicillin	rattlesnake capsule	recover
Jacobson 1989 [19]	49	F	AIDS	bacteraemia	ciprofloxacin	unknown	recover
Cone 1990 [14]	71	F	RA	bacteraemia/arthritis/gastroenteritis	ceftriaxone/chloramphenicol	folk remedy	recover
Cone 1990 [14]	72	M	metastatic melanoma	bacteraemia	aztreonam	rattlesnake capsule	recover
Casner 1990 [31]	30	M	AIDS/Kaposi’s sarcoma	bacteraemia/UTI	Baktar	rattlesnake capsule	recover
Caravalho1990 [25]	55	F	RA	endophthalmitis	cephalosporin	powder	recover
Woolf 1990 [27]	28	F	adenocarcinoma	peritonitis	ampicillin	capsule	death
Babu 1990 [30]	25	M	AIDS/Kaposi’s sarcoma	bacteraemia/gastroenteritis/UTI	ciprofloxacin	rattlesnake powder	recover
Noskin 1990 [15]	57	M	AIDS	bacteraemia	ampicillin	rattlesnake meat	recover
Kraus 1991 [21]	49	F	SLE	cholecystitis/bacteraemia	cephalothin/amikacin	rattlesanke capsule	recover
Kraus 1991 [21]	27	M	dermatomyositis	hip septic arthritis	ciprofloxacin	rattlesnake capsule	recover
Kraus 1991 [21]	34	F	SLE	knee septic arthritis	ciprofloxacin	rattlesnake capsule	recover
Kraus 1991 [21]	36	F	SLE	knee/shoulder septic arthritis/sepsis	ampicillin	rattlesnake capsule	recover
Kraus 1991 [21]	29	F	SLE	knee/shoulder septic arthritis/bacteraemia/UTI	ampicillin/amikacin	rattlesnake capsule	recover
Kraus 1991 [21]	24	F	leukaemia	bacteraemia	ciprofloxacin	unknown	recover
Kraus 1991 [21]	61	F	Primary biliary cirrhosis	vertebral osteomyelitis/UTI	ampicillin/amikacin	unknown	recover
Kraus 1991 [21]	25	F	SLE	UTI	TMP/SMX	NA	recover
Kraus 1991 [21]	70	M	DM, ALS	bacteraemia	TMP/SMX	rattlesnake capsule	recover
Sharma 1992 [29]	69	M	gastric cancer	peritonitis	cefotaxime/gentamicin	rattlesnake pills	recover
Cortes 1992 [26]	60	M	metastatic carcinoma	bacteraemia/pneumonia	aztreonam	rattlesnake meat	recover
Hoag 2005 [17]	47	F	AIDS	bacteraemia/pericarditis/gastroenteritis/UTI	ceftriaxone/ciproxin	pet chicken?	recover
Di Bella 2011 [4]	43	M	Hodgkin’s disease/ panhypoglobulinemia	gastroenteritis	ciprofloxacin	NA	recover
S. Kolker 2012 [9]	32	F	pemphigus	tibial abscess	ceftriaxone/ciproxin	iguana/snakes	recover

*F* female, *M* male, *SLE* systemic lupus erythematosus, *DM* diabetes mellitus, *HTN* hypertension, *HBV* hepatitis B, *AIDS* acquired immunodeficiency syndrome, *DCM* dilated cardiomyopathy, *CHF* congestive heart failure, *RA* rheumatoid arthritis, *ALS* amyotrophic lateral sclerosis, *UTI* urinary tract infection, *TMP/SMX* trimethoprim/sulfamethoxazole, *NA* not available

Patients in the present study were either elderly male or had various underlying conditions (Table 1) which would compromise their cell-mediated immunity. In the literature (Table 2), the most commonly reported comorbidities associated with *S. arizonae* infections were immunocompromised status, including connective tissue diseases under steroid therapy (40.9%), malignancy (27.3%) and acquired immunodeficiency syndrome (13.6%) [15, 17]. Our case series produced similar findings. Additionally, we identified three more associated host underlying conditions, including type 2 diabetes mellitus, liver cirrhosis, and peptic ulcer disease. Uncontrolled diabetes and liver cirrhosis have been shown to cause impairment of humoral- and cell-mediated immunity, which play important roles in clearing *Salmonella* [37]. Therefore, these two diseases would reasonably predispose to the development of *S. arizonae* infection. Moreover, patients with peptic ulcer diseases received acid suppressants to treat their diseases. Usage of acid suppressants would not only decrease the acidity of gastric juice, which in turn might result in intestinal bacterial overgrowth, facilitate bacterial translocation from the intestine and lead to infection via the gastro-intestinal route [38], but also reduce the gastric acid barrier with subsequent infection despite a lower inoculum of bacteria. Similar findings have been previously reported by Wu et al. [39].

A variety of clinical manifestations were displayed in our series, including enterocolitis, bacteraemia, vascular infection and localized infections. The rank order of infection syndromes in our reports was bacteraemia, intra-abdominal infection, and pulmonary infection. In contrast, bacteraemia [1, 7, 10, 13–15, 17–19, 21, 22, 26, 28, 30–32], intra-abdominal infection [4, 6, 10–12, 14, 17, 21, 24, 27, 29, 30, 32, 40] and bone or joint infection [8, 9, 11, 12, 14, 20, 21, 24] were the most common clinical manifestations in previously-reported patients (Table 2). Much fewer patients with bone and joint infection (16.7%) were noted in our present study. Interestingly, both of the two patients in our series diagnosed as *S. arizonae* related mycotic aneurysm with bacteraemia had a past history of hypertension. This is similar to the result by Wang JY et al*.*, who demonstrated that hypertension was the major factor predisposing to *S. choleraesuis* mycotic aneurysm [41]. Importantly, our study is the first one to demonstrate that soft tissue could be the infection focus of *S. arizonae*, which was observed in two of our patients. Overall, the difference in clinical manifestations between our study and prior reports might be due to the small number of patients enrolled in every study. Therefore, further study is needed to clarify whether geographic variance or other factors were associated with the difference of clinical syndromes.

All *S. arizonae* isolates collected in the present study were susceptible to all five of the recommended anti-Salmonella agents, including chloramphenicol, ciprofloxacin, trimethoprim/sulfamethoxazole, ampicillin, and ceftriaxone. These five antimicrobial agents have the ability to penetrate host cells, which is crucial in killing intra-cellular pathogens, such as *Salmonella*. All of our patients received at least one of these five agents as their definite therapy, among which third-generation cephalosporins were prescribed for the majority of patients (72.2%, Table 1). Instead, ampicillin was usually chosen as the backbone of the treatment modality (45.2%) in previously published articles (Table 2). Although the difference was not statistically significant (*p* = 0.1 by chi-square test), the crude in-hospital mortality rate was only 5.6% in the present study, which was much lower than that (10/44, 22.7%) of the prior reports (Table 2). In particular, 6 of the 10 fatal patients in prior reports received ampicillin as their treatment against *S. arizonae* infection. Therefore, a third-generation cephalosporin could potentially be a better choice for treating *S. arizonae* infection compared to ampicillin. However, further investigation is needed.

One patient in our study received piperacillin/tazobactam, which is not the recommended antibiotic as the definitive treatment against *Salmonellae* , and had a favorable outcome. Using piperacillin/tazobactam for *Salmonella* infection was rarely reported in previous articles. Bell SD demonstrated that minimal inhibitory concentration of piperacillin/tazobactam for *S. arizonae* isolated from this study was ≦4 μg/mL [4], implicating the bacterium susceptible to it in-vitro. Gerada et al*.* reported a liver transplant recipient with *Salmonella* related infectious aortitis and bacteraemia, who responded well to piperacillin/tazobactam treatment [42]. Thus, piperacillin/tazobactam might be considered as one of the therapeutic options for *S. arizonae* infection.

During the 1980s, *S. arizonae* infection became an important issue in public health due to the emergence of many severe infection cases, and its association with extensive use of rattlesnake-based products [10, 19, 28] mainly in areas with large Mexican-American populations in southwestern United States [1, 7, 13]. Approximately 70% of the 44 patients reported in previous studies (Table 2) mentioned a history of exposure to reptiles, especially snakes. Rattlesnake-based products are not common in Taiwan and we could not identify any specific animal contact history from the medical records of those patients. It is important to identify whether natural habitats of *S. arizonae* are present and whether *S. arizonae* infection is one of the zoonoses in Taiwan. Further studies are needed to identify the possible sources of this infection.

## Conclusions

Our study showed that *S. arizonae* infection, although uncommon, is present in Taiwan, an area outside of typical endemic areas. In addition to previously reported risk factors, usage of acid suppressants, such as proton pump inhibitors and H_2_ blockers, might also predispose to *S. arizonae* infection. To treat *S. arizonae* infection, third-generation cephalosporins might be more effective than ampicillin.

## Abbreviations

AIDS: Acquired immunodeficiency syndrome
ALS: Amyotrophic lateral sclerosis
ALT: Alanine aminotransferase
CHF: Congestive heart failure
CLSI: Clinical and laboratory standards institute
CRP: C-reactive protein
CYCH: Chia-Yi Christian hospital
DCM: Dilated cardiomyopathy
DM: Diabetes mellitus
F: Female
HBV: Hepatitis B
HTN: Hypertension
M: Male
NA: Not available
RA: Rheumatoid arthritis
SLE: Systemic lupus erythematosus
TMP/SMX: Trimethoprim/sulfamethoxazole
UTI: Urinary tract infection
WBC: White blood cell
